# Staphylococcal Panton-Valentine Leucocidin as a Major Virulence Factor Associated to Furuncles

**DOI:** 10.1371/journal.pone.0025716

**Published:** 2011-10-12

**Authors:** Lamine Baba-Moussa, Haziz Sina, Jean-Michel Scheftel, Brigitte Moreau, Dominique Sainte-Marie, Simeon O. Kotchoni, Gilles Prévost, Pierre Couppié

**Affiliations:** 1 Département de Biochimie et de Biologie Cellulaire, Université d'Abomey-Calavi, Cotonou, Benin; 2 Institut de Bactériologie, Unité EA-4438 Physiopathologie et Médecine, Strasbourg, France; 3 Service de Biologie Polyvalente, Centre Hospitalier de Cayenne, Cayenne, Guyane Française; 4 Service de Dermatologie, Centre Hospitalier de Cayenne et Institut Guyanais de Dermatologie Tropicale, Cayenne, Guyane Française; 5 Department of Biology, Rutgers University, Camden, New Jersey, United States of America; Instituto Butantan, Brazil

## Abstract

Panton-Valentine Leucocidin (PVL), one of the β-barrel pore-forming staphylococcal leucotoxins, is known to be associated to furuncles and some severe community pneumonia. However, it is still uncertain how many other virulence factors are also associated to furuncles and what the risk factors of furuncles are in immuno-compromised status of patients, especially the HIV (+) patients. In this paper, we use antigen immunoprecipitation and multiplex PCR approach to determine the presence of 19 toxins, 8 adhesion factors and the PFGE profiles associated to furuncles in three independent patient study groups of *S. aureus* (SA) isolates collected from the Cayenne General Hospital (French Guiana). The patient groups were made of: 16 isolates from HIV (−) patients, 9 from HIV (+) patients suffering from furuncles, and 30 control isolates from patients with diverse secondary infected dermatitis. Our data reveals that the majority (96%) of *SA* strains isolated from HIV patient-derived furuncles significantly produced PVL (*p*<10^−7^), whereas only 10% of *SA* strains produced this toxin in secondary infected dermatosis. A high prevalence of LukE-LukD-producing isolates (56 to 78%) was recorded in patient groups. Genes encoding clumping factor B, collagen- and laminin-binding proteins (*clfB*, *cna*, *lbp*, respectively) were markedly frequent (30 to 55%), without being associated to a specific group. Pulse field gel electrophoresis evidenced 24 overall pulsotypes, whereas the 25 PVL-producing isolates were distributed into 15 non clonal fingerprints. These pulsotypes were not specific PVL-producing isolates. PVL appears to be the major virulence factor associated to furuncles in Europe and in South America regardless of the immune status of the HIV patients.

## Introduction

*Staphylococcus aureus* (*SA*) are known to produce a large series of toxins [1], [2] and adhesion factors [3], [4] considered as virulence-associated factors. Among the virulence factors associated to clinical syndromes, only few were initially considered in most of original studies [5], [6], [7]. For example, the Panton Valentine leucocidin (PVL)-producing *SA* strains were successively associated to furuncles and carbuncles [5], [6], [8], [9] and/or to community pneumonia with lung abscesses [10], [11], [12].

PVL was initially identified because of the acute cytotoxicity of some culture supernatants of *SA* isolates from furuncles and staphylococcal necrotizing follicular infections of the skin [13], [14]. PVL is a skin-necrotizing toxin when intradermally injected into rabbits [15]. This bipartite toxin is an active pore-forming toxin against human polymorphonuclear cells, monocytes and macrophages [16]. However, the two protein components of PVL, LukS-PV and LukF-PV, may recombine with other related leucotoxins components, to generate new leucotoxins with specific cell spectra [17]. The newly formed toxins such as PVL and gamma-hemolysins (HlgA-HlgB or HlgC-HlgB sometimes called LukS-LukF) may be expressed at different levels [18] with highly variable binding constants to cells [19]. Sequence and three-dimensional structures of LukS-PV and LukF-PV have been determined [20], [21], and the interaction of LukS-PV with a membrane receptor was strongly suggested [19].

The origin and recurrence of furuncles as primary infections, and their severity in some patients remain unclear. This has been related to the nasal carriage of the bacterium [14], but this may suggest a role of innate immunity. Immunosuppressed HIV (+) patients develops frequently SA skin infections such as folliculitis and sometimes furuncle [22]. There is still no evidence of virulence factors in *SA* isolated from staphylococcal infections in HIV (+) patients. In addition, the possibility that immunodeficiency status of the patient may modify susceptibility to *SA* virulence has never been proved.

In this work we analyzed the toxin profile and adhesion factors of *SA* isolated from HIV positive and negative patients at the Cayenne General Hospital, the principal hospital in French Guiana, in South America, where HIV prevalence in adults was 1,6%. *SA* was isolated from independent furuncle and furuncle free patients. A comparative study of different *SA* toxins and adhesion factors was performed in order to establish the eventual association of virulence factors other than PVL, especially in HIV positive and negative patients with respect to secondary skin infections.

## Materials and Methods

### Ethics Statement

Samples collected from patients at French Guiana hospital center were anonymously sent to the University of Abomey-Calavi, Benin (West Africa) for microbial screening, while PFGE analysis were performed at Strasbourg University, Strasbourg, France. The authors had no access to the patient medical data, with the exception of anonymous clinical sample data such as ages, HIV status, skin infections. This work is mainly focused on the bacterium SA virulence factors and not on the patients, and was therefore exempted from ethical approval.

### Collected samples and clinical data

We collected *SA* isolates from 55 patients in Cayenne General Hospital (French Guiana). These patients were classified in three groups according to infectious origin: 30 patients with staphylococcal secondary skin infections (eczema: 13, wound: 7, prurigo: 6 and diverse dermatosis: 4), 16 HIV (−) patients with furuncles and 9 HIV (+) patients with furuncles. The HIV (+) status was discovered for 2 patients with furuncles by ELISA and Western blot, consecutively confirmed by dermatology consultation test (where HIV test was systematically proposed when a furuncle was diagnosed) and 7 patients were previously diagnosed as HIV (+) and they were regularly followed at hospital. The median of the CD4+ lymphocytes in HIV (+) patients was 225 U/mm3 (42–896). Four patients had CD4+ lymphocytes <200/mm^3^ and one <100/mm^3^. Among the HIV (+) patients, only one suffered of cerebellar toxoplasmosis.

The other patients were consecutively seen at dermatological consultation. In this study, only patients presenting lesions like furuncles or secondary skin infections of a pre-existing non infectious dermatosis due to *SA* were included. The demographic and clinical characteristics of patients were recorded before the bacteriological sampling on the lesion. The mean age of all 55 patients was 25.4 year old (1–69). It was 18.3 (1–55), 28.8 (2–56), 44.6 (20–69), respectively, for the patients with secondary infections, HIV (−) patients and HIV (+) patients. The sex ratio was 1.3.

### Susceptibility to antibiotics

Antibiograms for the isolates were obtained by the disk diffusion method (Bio-Rad-Diagnostic Pasteur, Marnes la Coquette, France). Isolates were inoculated and antibiotics were tested according to the 2010 Antiobiogram Committee of the French Society of Microbiology and the NCCLS recommendations [23]: Oxacillin (Oxa), Penicillin G (PeG), Vancomycin (Va), Teicoplanin (Tec), Kanamycin (Kan), Tobramycin (Tob), Chloramphenicol (Chl), Rifampicin (Rif), Fusidic acid (Fus), Gentamicin (Gm), Trimetroprim Sulfamethozolin (Sxt), Ofloxacin (OXF), Pristinamycin (Pt), Linezolide (Lzd) and Erythromicin (Ery) have been tested. Evaluation of methicillin resistance was performed by plating the strains on buffered Mueller-Hinton agar with 2% (wt/vol) NaCl at 37°C for 24 h.

### Phenotypic detection of toxins

#### Enterotoxins A, B, C, and D and TSST-1

*SA* cultures were carried out overnight in 3 mL of Brain-Heart (Difco) medium under shaking, and toxins were detected using the semi-quantitative reversed passive latex agglutination detection kits SET-RPLA™ and TST-RPLA™ (Oxoid, Basingstoke, England). These tests were reported to be sensitive and specific, although large amounts of enterotoxin E may be detected as low levels of enterotoxin A [24].

#### Bicomponent leucotoxins and epidermolysins

The different leucotoxins (PVL, LukE-LukD, LukM-LukF-PV, and the gamma-hemolysin proteins HlgA, HlgB, and HlgC), epidermolysins A and B were evidenced from culture supernatants after 18 h of growth in YCP medium [19] by radial gel immunodiffusion in 0.6% (wt/vol) agarose in 10 mM Hepes, 150 mM NaCl, pH 7.5 with component-specific rabbit polyclonal and affinity-purified antibodies [14]. For a better determination, gels were dried, stained in Coomassie blue, and destained in 10% (vol/vol) acetic acid.

### Genotype detection of toxins and adhesion factors

Presence of genes encoding entorotoxins SEE, SEG, SEH, SEK, SEL, SET, epidermolysin D (*etd*), Edin factors (*edinA*, *edinB*, *edinC*) and of genes encoding eight adhesion factors: Collagen Binding Protein (*Cna*), Fibronectin Binding Proteins A and B (*fnbA and fnbB*), Bone Sialoprotein Binding Protein (*bbp*), Clumping Factor B (*clfb*), Fibrinogen Binding Protein (*fib*), Elastin Binding Protein (*ebp*) and Laminin Binding Protein (*lbp*) was detected by Multiplex PCR (Table 1). Additionally, genes encoding PVL were also detected with oligonucleotides previously used for expression studies [18]. Total DNA was purified using QIAamp® DNA Mini Kit (Qiagen, GmbH, Germany) with a Gene Amp® PCR System 9700 (Perkin-Elmer, Norwalk, USA) and amplified in a total volume of 50 µl containing 25 pmoles of each primer, 50 ng of total DNA, 1.5 mM MgCl_2_, 200 µM of dNTP mixture, 1× PCR reaction Buffer and 5 units of FastTaq™ DNA polymerase (Roche Applied Science, GmbH, Germany). The thermal cycling conditions included an initial denaturation step (2 min at 92°C) followed by 35 cycles of amplification comprising three steps: 2 min denaturation for 92°C, 1 min annealing at 50°C, 2 min extension at 72°C. The reaction was terminated with 3 min extension at 72°C. PCR products were analysed by electrophoresis through 1.4% (wt/vol) agarose gel (Euromedex, Mundolsheim, France).

**Table 1:** Primers used for PCR detection of genes encoding virulence factors.

Oligonucleotides sequences	size pb/PCR set	accession n°
*edinA 1*: 5′-TCATAGAAGTATCTAATACTTCTTTAGCA-3′	604/2	M63917
*edinA 2* : 5′-TCCAACACGGTATTCTGTGCCTCTAGGTA-3′		
*edinb 1*: 5′-ACAGACTTAGTTGAAGCTACTAAATG-3′	522/5	AB057421
*edinb 2*: 5′-TGTCCCTGTAGGCAAAAGAACTTCTTG-3′		
*edinC 1*: 5′-AGGTCTTCCAGCTAATGCAGCTCCTT-3′	543/6	NC 003265
*edinC 2*: 5′-ACAGTTCAAAAGACAAAGAAGCTATT-3′		
*etd 1*: 5′-AATACATATGAAGAATCTGAAATTTTA3′	800/4	AB057421
*etd 2*: 5′-AAGTTATTCCATAATGATTAGAATGA-3′		
*lukE-lukD* 1: 5′-AATACTAATATTGAAAATATTGGTGATGG-3′	1882/8	Y13225
*lukE-lukD* 2: 5′-CTTAAATTTCGGGTTTAACTCCTCTATTTC-3′		
*see 1*: 5′-CTTACCGCCAAAGCTGTCG-3′	159/3	M21319
*see 2*: 5′-GTCCACTTGTAAATGGTAGCGAGAA-3′		
*seg 1*: 5′-AATTATGTGAATGCTCAACCCGAT-3′	408/3	AF064773
*seg 2*: 5′-CTTTAGTGAGCCAGTGTCTTGCTTTG-3′		
*seh 1*: 5′-CATCTACCCAAACATTAGCACC-3′	222/5	U11702
*seh 2*: 5-TAGAAATCAAGGTGATAGTGGCAA-3′		
*sek 1*: 5′-TGATACTCCTATAGCTAATCAACTACA-3′	300/3	U93688
*sek 2*: 5-ACATCAATCTCTTGAGCGGTAACA-3′		
*sel 1*: 5′-ACCAGAATCACACCGCTTAGAATAC-3′	422/2	AF217235
*sel 2*: 5′-TGGAATACTACACTCCCCTTATCAAAAG-3′		
*set1-1*: 5′-GAAGGTCTACAAGGCCAAAATGTCT-3′	363/6	BX571856
*set1-2*: 5′-TCAACACATCGCCCATGCGCTCGA-3′		
*bbp 1*: 5′-CGGCTAGTGATAATAAAGAAGTAGTG-3′	550/1	BA000018
*bbp 2*: 5′-CTTGTTGGAGCTGTAGCAACTGGTTT-3′		
*clfB 1*: 5′-ATTAGTGCAAACACAAACAGTGCG-3′	305/4	AJ224764
*clfB 2*: 5′-AGTTCCTTGCGCATTGGAAATCGT-3′		
*cna 1*: 5′-CGGGAGATATGCTACCAGAAGATA-3′	278/1	BX571857
*cna 2*: 5′-ATAGCCTTGTGGAATTGTTACATCA-3′		
*ebp 1: * *5*′-AGACCAATCAGAATTAGAACATCA-3′	378/4	U48826
*ebp2*: 5′-TCAGAAACTGTTGAATGCTCAGTGT-3′		
*fib 1*: 5′-AGCGGCAATAGGTATTACTACAACT3′	220/2	X72013
*fib 2*: 5′-CGAATGTACCATCGTTAAATTCAT-3′		
*fnbA 1*: 5′-TTAACTTGGGATAATGGTTTAGTTT-3′	273/7	AJ629521
*fnbA 2*: 5′-GCTGATGAATCCGTTTCTTCTATTG-3′		
*fnbB 1*: 5′-TGGAAGAAACTAAAGCGACAGGTAC-3′	317/1	AJ629502
*fnbB 2*: 5′-TTCTTTAAACGTATATCTAACTTTTC-3′		
*lbp 1*: 5′-TGGTGTATATGACTACAGTAAGTT-3′	410/5	AF065394
*lbp 2*: 5′-CGTTTGTAGCAACAGCAATATCAGC-3′		

Other virulence factors have been detected by multiplex PCR onto purified total DNA (Qiagen). Presence of genes encoding enterotoxins E, G, H, K, L, T, epidermolysin D (Etd), genes encoding Edin A and EDIN B and the genes encoding seven adhsesion factors (Cna, FnbA, FnbB Bbp, Clfb, Fib, Ebp and Lbp) was checked in 8 set in function of base size.

### Pulse Field Gel Electrophoresis (PFGE)

*SA* strains at mid-exponential growth in 25 ml of TY (1.6% [w/v] BioTrypcase, 1% [w/v] yeast extract, 0.5% [w/v] NaCl,) were embedded in agarose plugs as described previously [25]. After lysis of plugs by proteinase K (Sigma) for 18 h at 56°C, plugs were washed and stored at 4°C in 10 mM Tris-HCl, 1 mM EDTA (pH 8.0). Plugs (agarose plugs, 2.5 by 5.0 by 1 mm) were equilibrated in 300 µL of restriction enzyme buffer for 30 min at 4°C. DNA macro-restriction was then accomplished in 60 µL with 10 U of *Sma* I (New England Biolabs, Beverly, Mass.) for 4 h at room temperature. Electrophoresis was performed at 12°C with a Beckman Geneline™ Transverse Alternating Field Electrophoresis (TAFE) system at 150 mA in 0.5× TAFE system buffer (TAFE buffer is 0.2 M Tris, 10 mM free acid EDTA, and 87 mM CH_3_COOH [pH 8.2]) in a 1% (w/v) agarose gel. Electrophoresis of *Sma*I-fragmented DNA was carried out with 2-s pulses for 1 h, followed by 14-s pulses for 1 h, 12-s pulses for 1.5 h, 10-s pulses for 2.5 h, 8-s pulses for 6 h, and 6-s pulses for 6 h. Bacteriophage lambda PFG Marker (New England Biolabs) was used as a molecular marker. The gels were stained with ethidium bromide and photographed under UV trans-illumination. Pulsotypes were compared and classified in a dendrogram using the Dice coefficient and the unweighted pair group method with arithmetic mean clustering provided by Molecular Analyst™ (version 1.5) and Fingerprinting™ (version 1.12) softwares (Bio-Rad, Ivry sur Seine, France). Isolates that differed by no more than three fragments were considered to be subtypes of a given clonal type.

### Statistical analysis

For comparison tests of positive isolates of each patient group, we used the Student T test, and the Fischer's test for lower number series (SPSS Ltd software, version 10.1). P<0.05 was considered statistically significant.

## Results

### Susceptibility to antibiotics

In this study, no isolate was resistant to oxacillin or gentamicin. However, production of β-lactamase was observed for all isolates of the three groups with the exception of one isolate of the control group. In addition, the isolates were generally sensitive to all tested antimicrobials with the exception of few SA that were resistant to fusidic acid, *i.e.* 9 of the 10 fusidic acid resistant isolates were from the control group, or to erythromycin, *i.e.* 9 of the 11 erythromycin resistant isolates were also issued from the control group.

### Comparative analysis of toxins production

The presence of the staphylococcal toxins in the different groups is detailed in the Table 2. A great majority (96%) of the *SA* strains isolated from furuncles regardless of status of the HIV patient produced PVL, while only 10% of the isolates originated from secondary infected dermatosis produced this toxin (Table 2). This difference between furuncles and non-furuncles populations was significant (*p*<10^−7^). All the strains isolated from furuncles of HIV (−) patients produced PVL, while it is produced by 89% of the isolates obtained from furuncles of HIV (+) patients. Only PVL-producing isolates were also detected for the presence of corresponding genes, conversely to the LukE/LukD leucotoxin. In fact, the *lukE/lukD* locus was detected in most *SA* isolates (92%), but the effective production of the corresponding LukE/LukD leucotoxin was observed only for 64% of isolates obtained from furuncles and for 76% of those from secondary infected dermatosis (Table 2). Therefore, no noticeable difference between groups could be drawn in this study. This result illustrates heterogeneity between *SA* isolates from our sample collection in regards to the occurrence of LukE/LukD production [24], [26], [27]. Epidermolysin A (ETA) is produced by 16% of isolates from secondary infected dermatosis. The presence of enterotoxins A and G yielded 23% and 16% in the group of secondary infections respectively, while only 4% and 16% in the cumulated groups of furuncles, respectively. Finally, no more than 40% (12/30) of the secondary dermatitis- and no more than 36% (9/25) of the furuncle-originated isolates produced one at least of the most encountered superantigens.

**Table 2:** Production of toxins and identification of genes encoding toxins in *Staphylococcus aureus*.

Toxins and genes encoding toxins	Infected dermatosis (n = 30)		Furuncles (n = 25)	
		All	HIV (−) (n = 16)	HIV (+) (n = 9)
SEA	7 (23.3%)	1 (4.0%)	1 (6.3%)	0
SEB	3 (10.0%)	5 (20.0%)	2 (12.5%)	3 (33.3%)
SEC	4 (13.3%)	4 (16.0%)	1 (6.3%)	3 (33.3%)
SED	2 (6.7%)	1 (4.0%)	1 (6.3%)	0
TSST1	1 (3.3%)	0	0	0
ETA	5 (16.7%)	0	0	0
ETB	0	1 (4.0%)	1 (6.3%)	0
PVL	3 (10.0%)	24 (96.0%)	16 (100.0%)	8 (88.9%)
LukE/LukD	23 (76.7%)	16 (64.0%)	9 (56.3%)	7 (77.8%)
*lukE-lukD*	29 (96%)	22 (88%)	13 (81.5%)	9 (100%)
*etd*	0	0	0	0
*see*	0	0	0	0
*seg*	5 (16.7%)	7 (28.0%)	2 (12.5%)	5 (55.6%)
*seh*	0	0	0	0
*sek*	0	0	0	0
*sel*	0	0	0	0
*set*	0	0	0	0
*edinB*	0	0	0	0
*edinC*	0	0	0	0
*edinA*	0	0	0	0

The majority of *SA* strains isolated from HIV patient-derived furuncles significantly produced PVL (*p*<0.05), whereas only 10% of *SA* strains produced this toxin in secondary infected dermatosis. A high prevalence of LukE-LukD-producing isolates (56 to 78%) was recorded in patient groups.

### Comparative analysis of the presence of adhesion factors

The distribution of 8 genes encoding adhesion factors were examined (Table 3). Gene encoding *FnBA* was carried out in all tested isolates. In the groups of the strains isolated from furuncles and the secondary infected dermatitis, no isolate carries genes encoding *Fib*, *Cna*, *FnBb*, *Bbp*. The presence of *ClfB* seemed to be linked to the presence of *Lbp*, since all the strains carrying *clfB* also carried *lbp*. Ebp is produced by 48% of the isolates from furuncles, while it is produced by 30% in the case of non-infected dermatitis. Concerning the two HIV (+) and HIV (−) patient groups, the genes encoding the adhesion factors ClfB, Lbp, and EBP are present respectively at 6/16, 6/16 and 7/16 for HIV (−) group and 4/9, 4/9 and 5/9 for HIV (+) group.

**Table 3:** Presence of genes encoding adhesion factors of *Staphylococcus aureus*.

Gene encoding adhesion factors	Infected dermatosis (n = 30)		Furuncles (n = 25)	
		All	HIV (−) (n = 16)	HIV (+) (n = 9)
*bbp*	0	0	0	0
*cna*	0	0	0	0
*clfb*	16 (53.3%)	10 (40%)	6 (37.5%)	4 (44.4%)
*ebp*	9 (30%)	12 48%)	7 (43.8%)	5 (55.6%)
*fib*	0	0	0	0
*fnbA*	30 (100%)	25 (100%)	16 (100%)	9 (100%)
*fnbB*	0	0	0	0
*lbp*	16 (53.3%)	10 (40%)	6 (37.5%)	4 (44.4%)

Genes encoding clumping factor B, collagen- and laminin-binding proteins (*clfB*, *cna*, *lbp*, respectively) were markedly frequent (30 to 55%), without being associated with a specific group.

### Pulsed Field Gel Electrophoresis (PFGE)

**Figure 1:**
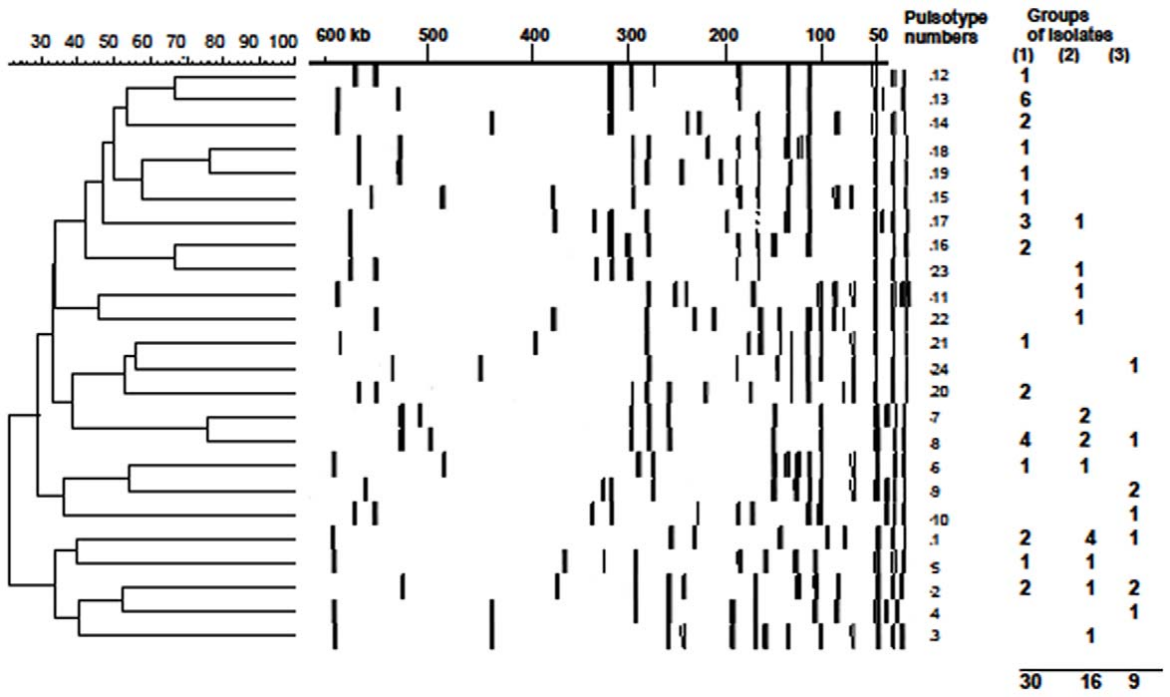
Pulsed field gel electrophoresis (PFGE) dependent dendogram of isolated *Staphylococcus aureus*. Pulsed field gel electrophoresis (PFGE) proves no specific clonal relationship between PVL-producing isolates issued from furuncles or secondary dermatosis. The similarity of the different pulsotypes was established by using Molecular Analyst™ software. Twenty four pulsotypes corresponded to the 55 isolates and their distribution is given according to the groups of isolates issued from secondary infected dermatosis (1), furuncles from HIV (−)(2) or HIV (+)(3) patients.

Pulsed field gel electrophoresis defined 24 *Sma*I-pulsotypes differing by more than two DNA fragments for the 55 different isolates, which therefore, exclude clonal relationships between isolates (Figure 1). Among the different pulsotypes, which account for a diverse collection of SA genotypes in French Guiana, some were identified in both furuncles and secondary pyodermites isolates. Therefore, PVL-producing isolates cannot be referred to as a specific category of pulsotype. According to the isolates, 9 to 14 defined DNA fragments of lengths comprised between 20 kb and 600 kb after *Sma*I restriction. Six of these pulsotypes were displayed by isolates belonging to different groups including secondary dermatitis. The most represented pulsotypes n° 1, 8, 13 are composed of 7, 6, and 6 isolates respectively. The diversity of the pulsotypes accounts for an endemic polymorphic SA isolates in French Guiana and reinforces the PVL as a main causative agent of furuncles.

## Discussion

This study reinforces PVL as probably the major staphylococcal virulence factor in furuncles as it is produced by almost all *SA* isolates, i.e. 96% from furuncles and (10%) (*p*<10^−7^) from control group constituted of *SA* isolates from non furunculous skin infections. These results are comparable with those previously obtained in Europe [28], [29], [30]. In Strasbourg (France), 85.7% of *SA* strains isolated from a series of 35 furuncles produced this toxin, while it was produced by less than 2% of strains issued from healthy patients or patients with other staphylococci affections [8], [14]. In former studies, PVL production from routine isolates represented 3 to 12% of isolates according to collections [14], [31]. Other authors established the association with furuncles at 93% within a series of 30 furuncles [11]. Together with other research group, we have previously noticed that isolates obtained simultaneously from different furuncles, or from different episodes or in a same fratry were comparable. However, they are less comparable if they are isolated from individuals, except when they were obtained from outbreaks [6], [32], [33]. All together, the absence of clonal relationships between these American isolates confirms the significance of the association of PVL with furuncles.

A furuncle is characterized by necrosis of body hair follicles and adjacent tissues. In rabbit skin, PVL induces necrosis after intradermal injection [14], [15]. The *SA* PVL-producing strains were also associated to severe primitive staphylococci pneumonia with high mortality rate, and with multiple consecutive lung abscesses to metastastic diffusion from furuncles [10]. However, it could not be excluded from any of these studies that another virulence factor may be also associated with PVL. We almost excluded the role of superantigens and other major toxins, while no specific adhesion factor can be suspected. In fact, despite the presence of such a factor, adhesion of *SA* provides a benefit to the bacterial colonisation, but a specific adhesion remains questionable.

Our study did not found a significant difference (*p* = 0.36) between the PVL produced by *SA* strains responsible for the furuncles regardless of the status of HIV patient considered: HIV (+) (89%), HIV (−) (100%). Among HIV (+) patients, the chronic nasal carriage of *S. aureus* is more frequent [34]. The frequency of *SA* infections increased with the decreasing of immunity. Severe *SA* infections are generally described among the patients with CD4+ lymphocytes less than 100 cells/mm^3^ [22], [35]. In the case of a chronic nasal carriage, the risk for *SA* infection rose to 20% per year among patients with CD4+ <100 cell/mm3 [35]. The frequency of *SA* skin infections increased with the immunodeficiency. At an advanced stage of the disease, 20% of the patients may be concerned by a *SA* skin infection [22]. The furuncles or carbuncles are within the first reported *SA* skin infections among the HIV (+) patients [35], [36]. Like in HIV (−) patients, in HIV (+) patients the principal bacterial factor associated with furuncle is PVL-producing *SA* infecting a follicle body hair. This data argued for the association of PVL-production with some necrotic lesions like furuncles and carbuncles.

For HIV (+) patients and others, furuncle is a sign of the presence of a virulent *SA*-strain, which produces PVL. In the context of advanced immunodeficiency, this infection must be particularly cured as well as the bacterial carriage, because of the risk of consecutive septicaemia due to this kind of strain. Recently, the emergence of Methicillin Resistant *SA* (*MRSA*) isolates producing Panton-Valentine Leucocidin (PVL) have been observed [37], [38], [39], thus highlighting additional seriousness of potential complication.

In our study, the *SA* strains isolates involved in furuncles did not harbour any specific resistance to antibiotics. The high frequency of LukE/LukD-producing isolates observed in the groups of strains (55 to 85%), may be due to an endemic situation, which should be further confirmed. A markedly high frequency of some adhesion factors *Clf*, *Lbp* and *Ebp* does not seem to be associated to any particular group of isolates.

In conclusion, and like in Europe, we demonstrate here a strong association between SA-producing PVL isolates in South America and necrotic skin lesions such as furuncles. This association is similar in HIV (+) and in HIV (−) patients. It remains unclear whether such skin lesions might be considered as risk markers in exposed patient's populations. Absence of clonal relationships between SA strains isolated from furuncles is an important argument for a causal relationship between capacity of PVL production by the SA and the development of furuncles.
